# Rapid quantification of plant-powdery mildew interactions by qPCR and conidiospore counts

**DOI:** 10.1186/1746-4811-8-35

**Published:** 2012-08-31

**Authors:** Ralf Weßling, Ralph Panstruga

**Affiliations:** 1 Department of Plant-Microbe Interactions, Max-Planck-Institute for Plant Breeding Research, Carl-von-Linné-Weg 10, Cologne, 50829, Germany; 2 Institute for Botany, Unit of Plant Molecular Cell Biology, RWTH Aachen University, Aachen, 52056, Germany

**Keywords:** *Arabidopsis thaliana*, Conidiospores, *Golovinomyces orontii*, Powdery mildew, Quantification, qPCR

## Abstract

**Background:**

The powdery mildew disease represents a valuable patho-system to study the interaction between plant hosts and obligate biotrophic fungal pathogens. Numerous discoveries have been made on the basis of the quantitative evaluation of plant-powdery mildew interactions, especially in the context of hyper-susceptible and/or resistant plant mutants. However, the presently available methods to score the pathogenic success of powdery mildew fungi are laborious and thus not well suited for medium- to high-throughput analysis.

**Results:**

Here we present two new protocols that allow the rapid quantitative assessment of powdery mildew disease development. One procedure depends on quantitative polymerase chain reaction (qPCR)-based evaluation of fungal biomass, while the other relies on the quantification of fungal conidiospores. We validated both techniques using the powdery mildew pathogen *Golovinomyces orontii* on a set of hyper-susceptible and resistant *Arabidopsis thaliana* mutants and found that both cover a wide dynamic range of one to two (qPCR) and four to five (quantification of conidia) orders of magnitude, respectively. The two approaches yield reproducible results and are easy to perform without specialized equipment.

**Conclusions:**

The qPCR and spore count assays rapidly and reproducibly quantify powdery mildew pathogenesis. Our methods are performed at later stages of infection and discern mutant phenotypes accurately. The assays therefore complement currently used procedures of powdery mildew quantification and can overcome some of their limitations. In addition, they can easily be adapted to other plant-powdery mildew patho-systems.

## Background

Powdery mildew fungi are widespread pathogens of agronomic importance. These parasitic Ascomycetes can infect more than 10,000 plant species, including many economically relevant crops and ornamentals [1]. All powdery mildew fungi are obligate biotrophic pathogens and therefore require living host cells for growth and reproduction [2].

Most powdery mildew fungi complete their life cycle on the leaf surface and obtain nutrients only from epidermal cells. Different stages of infection can be discriminated by light microscopy, which start with the germination of the spore and the generation of an appressorium, a specialized infection structure that differentiates at the tip of the germ tube. The appressorium facilitates the penetration of the host cuticle and cell wall and, if successful, the generation of the haustorium, the primary feeding structure of the fungus. Subsequently, secondary hyphae are formed, spread epiphytically and secondary haustoria are established in neighbouring host cells. Four to seven days post inoculation (dpi) abundant epiphytic conidiation is apparent, producing the characteristic white powdery mildew pustules [2].

The dicotyledonous model plant *Arabidopsis thaliana* is susceptible to infection by four different powdery mildew species: *Erysiphe cruciferarum*, *Golovinomyces cichoracearum*, *Golovinomyces orontii* and *Oidium neolycopersici* (see [2] for review). In recent years, these interactions have been used to reveal important components of the Arabidopsis immune system. Among them were several genetic factors governing disease susceptibility and resistance [3-6]. The interaction of Arabidopsis and powdery mildew fungi thus evolved as a model to study plant-biotroph interactions [2].

Currently, the quantification of powdery mildew infection on plants is based on three major methods that all have certain limitations: macroscopic categorization and microscopy-based penetration and conidiophore counts [3,7,8]. For crude categorization, disease symptoms can be scored by eye at late stages of pathogenesis (7–14 dpi) and ratings assigned based on the severity of disease symptoms [6,8]. While this method is quick and suitable for high throughput, it is prone to subjectivity, relies on equal inoculation densities and can only reveal strong differences in colonization that are readily visible to the naked eye. Assessment of host cell entry by penetration counts is a quantitative way to measure powdery mildew infection [3,9], but this method is limited to differences in susceptibility that are already manifested at early stages of fungal pathogenesis. In addition, it requires time-consuming staining and mounting steps of multiple microscopic samples and the subsequent assessment of hundreds of interaction sites. Finally, conidiophore counts have been used to characterize small mutant sets in detail [3,7,8]. This method requires tight control of inoculation density to ensure the presence of single fungal colonies, and, similar to penetration counts, necessitates tedious staining and mounting of multiple microscopic samples. In addition, hyper-susceptibility of genotypes can sometimes not be resolved by this technique [8]. Based on their microscopic nature involving staining of specimens, the latter two methods are unsuitable for the analysis of large sample contingents such as mutant collections or segregating populations. A microscopy-based quantification method for the analysis of intermediate stages (fungal colonies) also has been developed, but either requires tedious manual micro-photographic time series [10,11] or expensive automated microscopy systems [12].

Here we report the development of two assays that quantify powdery mildew infection on the basis of either qPCR or spore counts. While one of these techniques (spore counts) also quantifies asexual reproduction (conidiation), the other (qPCR) is suitable to follow infection over time and can thus resolve the kinetics of fungal pathogenesis. We validated the proposed procedures by the employment of hyper-susceptible and resistant Arabidopsis mutants. These fast and quantitative procedures, which can be performed at the seedling stage, are suitable for medium- to high-throughput analysis and will help to reveal comparatively small differences in powdery mildew disease susceptibility of natural accessions and mutant plant lines.

## Results and discussion

The recent advancements in the field of powdery mildew research [13] call for the development of new, quantitative, medium- to high-throughput methods for the quantification of the powdery mildew infection. We therefore attempted to develop two complementary methods that would allow the sensitive detection of rather small differences in susceptibility of plants to powdery mildew infection. We selected the interaction between Arabidopsis and *G. orontii* as an experimental system since a range of resistant and hyper-susceptible mutants are available for this plant-microbe combination. We focused on a qPCR-based and a spore count-based method as these procedures have been successfully applied to other plant-pathogenic microorganisms before [14-18]. Yet, to the best of our knowledge, these methods have not been adapted to the quantification of powdery mildew pathogenesis.

**Figure 1:**
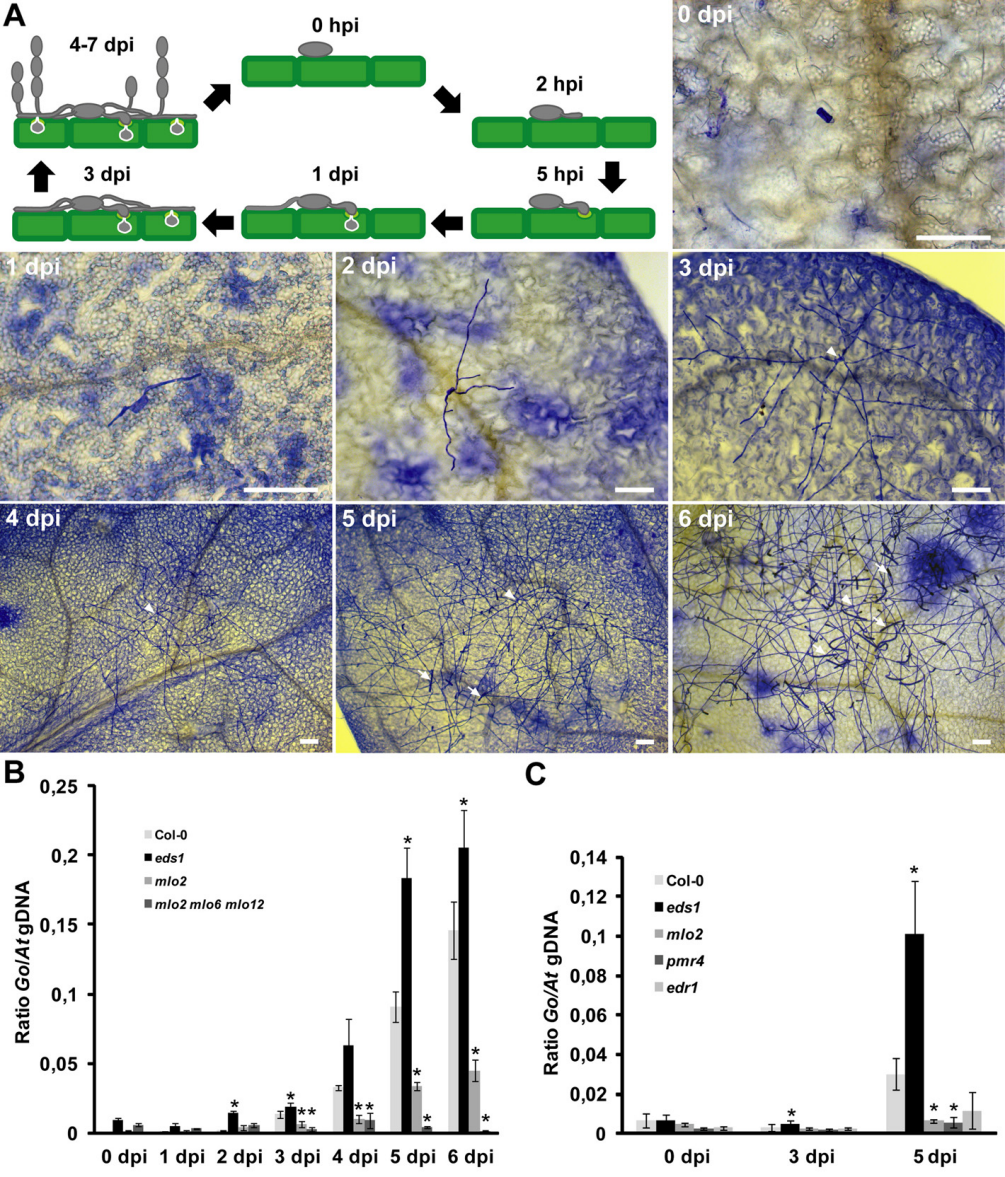
**Powdery mildew disease progression on Arabidopsis seedlings.** (**A**) Schematic overview and microscopic images of powdery mildew disease progression on Col-0 seedlings. Samples were harvested at indicated time points and stained with Coomassie Brilliant Blue. Arrows indicate conidiospore chains and arrowheads point to the initial spore. hpi, hours post inoculation (**B**) qPCR analysis of a time series of powdery mildew infection on Col-0 wild type, *eds1*, *mlo2* and *mlo2 mlo6 mlo2* seedlings. Ratios of *G. orontii* to Arabidopsis gDNA were determined by qPCR with primers R189/R192 and R193/R194, respectively. Bars represent the mean ± standard deviation of three technical replicates from a DNA sample of ten pooled seedlings grown in five different pots (two seedlings/pot used). (**C**) qPCR analysis of powdery mildew infection on Arabidopsis mutants that show powdery mildew-induced cell death. Representative time points of infection on Col-0 wild type, *eds1*, *mlo2, pmr4* and *edr1* seedlings were used. Ratios of *G. orontii* to Arabidopsis gDNA were determined by qPCR with primers R189/R192 and R193/R194, respectively. Bars represent the mean ± standard deviation of three DNA samples (each derived from ten pooled seedlings grown in five different pots) with three technical replicates each. Asterisks indicate statistically significant differences to Col-0 in two-tailed Student’s t-test (p <0,05). Schematic overview in (**A**) is courtesy of Justine Lorek. Scale bars in (**A**) are 100 μm. Data shown are representative of three independent experiments (see Additional file 2 for data of a second experiment).

First, we conducted a cytological analysis of the *G. orontii* infection on wild type (ecotype Col-0) seedlings (Figure 1A). The use of seedlings allows a more rapid screening of phenotypes (2–3 week old instead of 4–5 week old plants) and produces an averaging effect due to the use of many individuals (up to a hundred) per genotype and biological replicate. The susceptibility of a genotype can vary based on environmental conditions like pot humidity and averaging across pots can help to control this effect. To apply the inoculum we used a settling tower as this method allows a uniform and controlled inoculation and can be used efficiently for larger amounts of genotypes [6,8,19]. Inoculation densities were kept low (~750 spores/cm^2^) to discern single powdery mildew colonies on the leaves. 

The life cycle of *G. orontii* can be separated into several distinct stages, but so far its timing has not been characterized on Arabidopsis seedlings. We found that at 1 dpi most primary haustoria had been formed and growth of secondary hyphae began (Figure 1A). Hyphal development continued slowly until 2 dpi and increased rapidly after the formation of secondary haustoria at 3 dpi (Figure 1A). The Col-0 ecotype is highly susceptible to *G. orontii* and conidiophores therefore sometimes already formed at 4 dpi. Subsequently, the number of conidiophores increased and numerous growing chains of conidiospores were observed at 5 and 6 dpi. Our observations of the infection process on seedlings are in line with previous reports on *G. orontii* infections of 4–5 week old Arabidopsis plants [2,20]. The use of seedlings therefore faithfully reflects the timing of the natural infection process on mature plants.

### qPCR-based quantification of *G. orontii* infection

Methods on the basis of qPCR have been developed for biomass quantification of many plant-pathogenic microorganisms [15-17]. For this procedure, the quantitative extraction of pure genomic DNA as well as the efficient and specific amplification of target sequences is key. We therefore used a phenolic extraction technique for genomic DNA isolation that was previously found to allow quantitative DNA isolation from both fungal and bacterial plant pathogens [16]. The protocol was modified by introducing a disruption step of frozen material, as direct disruption of fresh seedlings was inefficient in our hands. We then derived a series of qPCR primers from arbitrarily chosen genes to amplify either *G. orontii* or Arabidopsis genomic sequences and tested them for amplification efficiency. For efficient primer pairs we optimized the annealing temperatures and primer concentrations to obtain most specific PCR results. Primer dimers were not detected for any of the primer pairs by either melting curve analysis or agarose gel electrophoresis. Subsequently, we performed a 5-fold dilution series of genomic DNA from heavily infected Col-0 tissue (harvested at ~14 dpi) to generate a standard curve for primer efficiency calculation across the dynamic range (see Additional file 1: Figure S1A). All tested primer pairs were found to have high amplification efficiencies of 90 to 100% (Table 1). For the *G. orontii* primer pairs, marginal background amplification of unspecific products was detected on an uninfected Col-0 control sample (see Additional file 1: Figure S1B). This did not affect the procedure since the unspecific amplification was associated with Ct values (>35) that were outside the range used in the subsequent experiments (ca. 25–35). We used the amplification of *G. orontii*-derived amplicons relative to products obtained from Arabidopsis genomic DNA for quantification of fungal biomass. This measure controls for variation in both sample harvesting and the efficiency of DNA isolation. 

**Table 1:** Primer sequences and amplification efficiencies

**Primer**	**Sequence**	**Target**	**Accession#**	**Amplicon in bp**	**Efficiency in%**
R189	GAATCCACCCATACCACCAG	RNA-binding (RRM/RBD/RNP motifs) family protein	At3g21215	114	95
R192	GAGGAGGAGGATGGTGATGA				
R243	AAGCACCTCCTGCTGTTCAT	Glyceraldehyde-3-phosphate dehydrogenase of plastid 2	At1g16300	125	91
R244	CTTTCCACTGCTCCTTGACC				
R193	TCGCCGCTATATTTGGAGTC	Plasma membrane ATPase 1	Go_V1_Contig3757	90	90
R194	CTGGGTCAGATGGTTCACCT				
R263	TCTTGGTGGCACGAATGAC	GDSL-like lipase	Go_V1_Contig76	92	100
R264	AGTGCGAGAGTGGGACAGAC				

**Figure 2:**
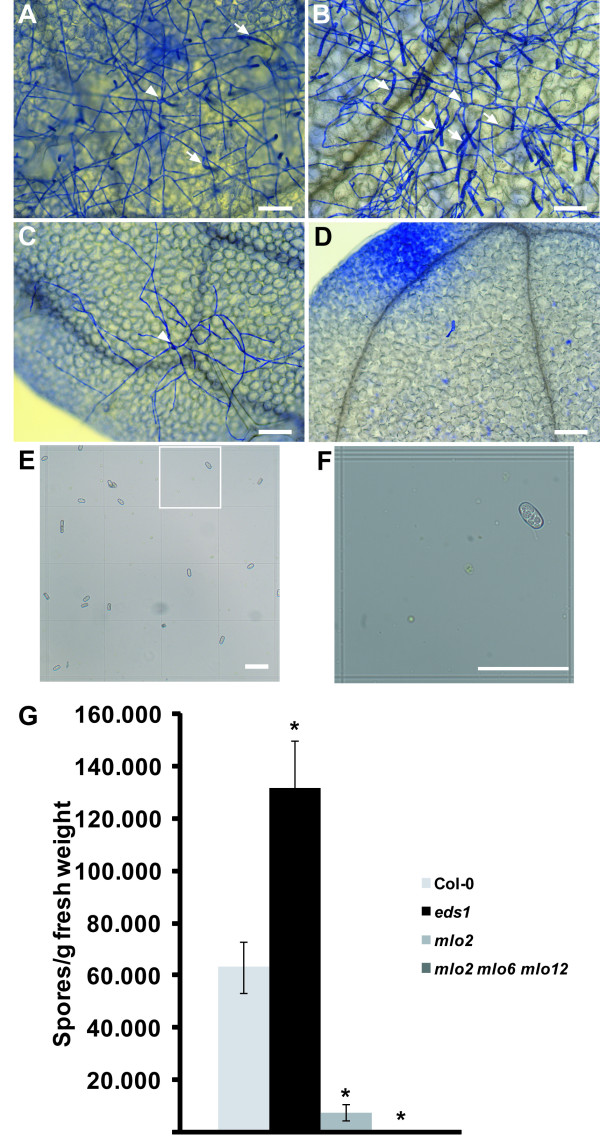
**Analysis of powdery mildew infection by spore counts. ***G. orontii* infected leaves were harvested at 5 dpi from Col-0 wild type (**A**), *eds1* (**B**), *mlo2* (**C**) and *mlo2 mlo6 mlo2* (**D**) seedlings and stained with Coomassie Brilliant Blue. Arrows indicate conidiospore chains and arrowheads point to the initial spore. (**E, F**) Brightfield image of isolated spores in the haemocytometer. (**F**) is a close-up of the indicated area in (**E**). (**G**) Spore counts of indicated genotypes at 6 dpi normalized to seedling fresh weight. Bars represent the mean ± standard deviation of three samples (500 mg of seedlings each) from one experiment counting eight fields/sample. Asterisks indicate statistically significant differences to Col-0 in two-tailed Student’s t-test (p <0,05). Scale bars in (**A**-**F**) are 100 μm. Data shown are representative of three independent experiments (see Additional file 2 for data of a second experiment).

**Figure 3:**
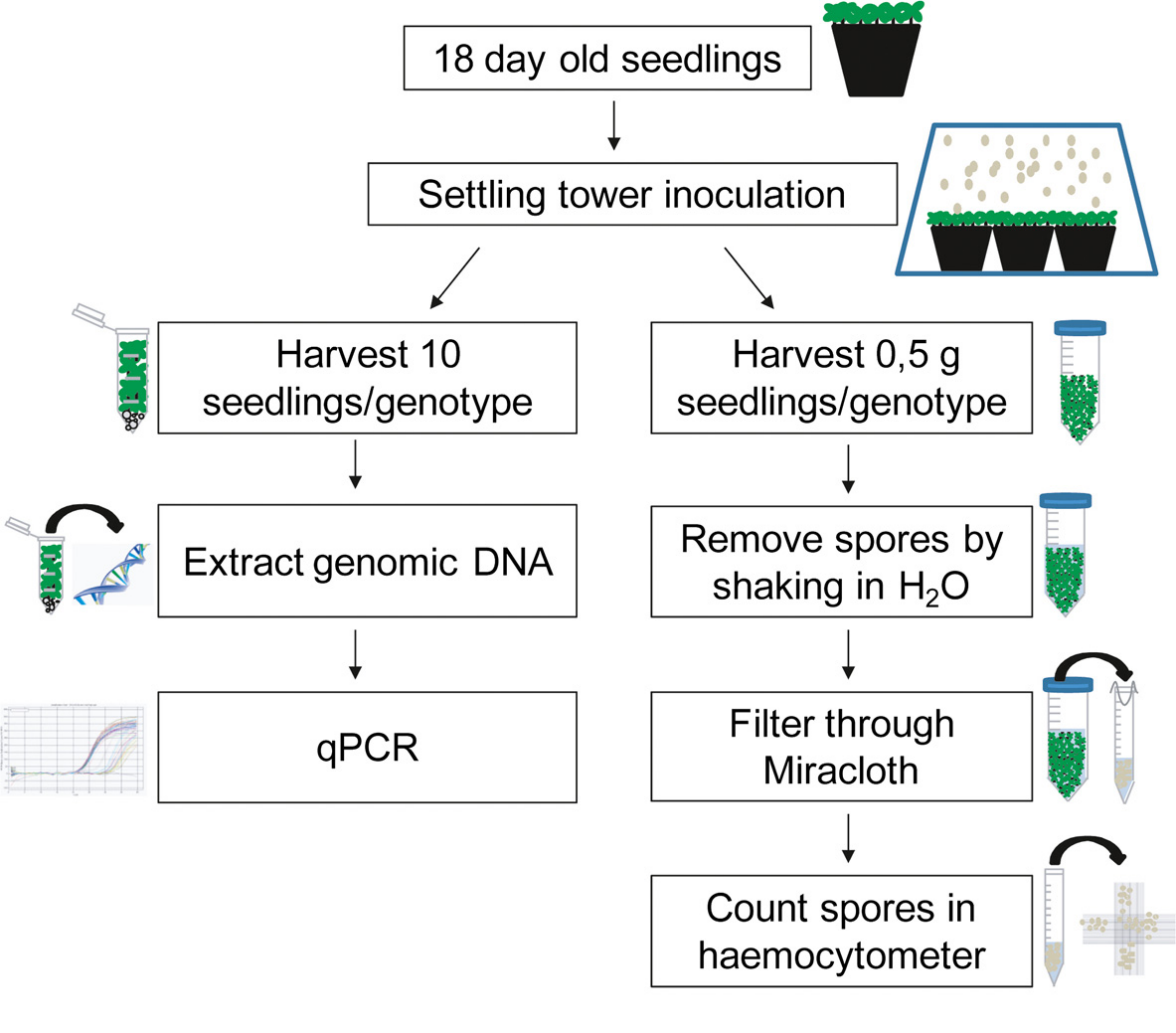
**Schematic overview of developed methods.** Simplified view of the workflow of the qPCR- and spore count-based powdery mildew quantification procedures. For further details see main text.

To determine the dynamic range of our qPCR assay we used an infection time series of Col-0 wild type, *eds1*, *mlo2* and *mlo2 mlo6 mlo12* triple mutant plants (Figure 1B). Col-0 plants are very susceptible to *G. orontii* as penetration rates of up to 90% are typically observed [3]. The *eds1* (*enhanced disease susceptibility1*) mutant is compromised in both salicylic acid-dependent and -independent defense signaling pathways and thus hyper-susceptible to *G. orontii* infection [4,21]. Mutations in particular *Mildew Resistance Locus O (MLO)* genes confer quantitative and additive penetration resistance to *G. orontii*, with penetration rates of ~40% in *mlo2* single mutants and ~1% in the *mlo2 mlo6 mlo12* triple mutant [3,9]. Using primers R189/R192 (At3g21215) and R193/R194 (*G. orontii* Plasma membrane ATPase 1; see Table 1) in the qPCR analysis, relative *G. orontii* DNA abundance increased slightly until 2 dpi in the wild type and subsequently increased strongly, which reflects the microscopical observations (Figure 1A). Differences between genotypes became first detectable at 3 dpi and increased until 5 dpi. For the hyper-susceptible mutant *eds1*, we repeatedly detected a saturation effect at 6 dpi (Figure 1B and Additional file 2: Figure S2A). The *mlo2 mlo6 mlo12* plants show complete resistance to *G. orontii* penetration and therefore allow no hyphal expansion (Figure 2D and [3,9]). Differences between ratios in the time course of this genotype are therefore probably due to small differences in inoculation densities as well as DNA degradation in dead and dying spores. This also leads to a dilution effect at later (4–6 dpi) time points of infection. At 5 dpi, approximate 5:2:1 ratios of relative *G. orontii* abundance of *eds1* to wild type to *mlo2* were repeatedly obtained (Figure 1A, Additional file 2: Figure S2A). Similar results were obtained with primer pairs R243/R244 (At1g16300) and R263/R264 (Additional file 3). 

The qPCR data correlate well with the microscopic analysis of the genotypes at 5 dpi (Figure 2A-D). At this time, *eds1* plants display enhanced hyphal growth and more conidiation than the wild type. The *mlo* mutants show either strongly reduced (*mlo2*) or no hyphal growth (*mlo2 mlo6 mlo12*). Previous reports on the *mlo* mutants used penetration as well as conidiophore counts to assess differences in susceptibility among mutants and the wild type [3,9]. While *mlo2* and *mlo2 mlo6 mlo12* plants differed considerably in penetration resistance, conidiophore counts were similar (both close to zero). The qPCR-based quantification of fungal biomass at 5 dpi therefore accurately reflects the impaired fungal development on enhanced disease resistance mutants. The method does, however, not reproducibly resolve differences in penetration rates at early time points, e.g. at 1 or 2 dpi (compare data in Figure 1A, Additional file 2: Figure S2A and Additional file 3). In the *eds1* mutant, penetration rates are indistinguishable from wild type and hyper-susceptibility only becomes evident at later stages of infection [4,22]. Our qPCR assay can detect the hyper-susceptibility phenotype of this mutant already at 3 dpi, demonstrating the power of the method. Overall, 5 dpi is most suitable for the qPCR-based comparative quantification of *G.orontii* infection. At this time point differences in fungal biomass between genotypes are most pronounced and saturation effects are not yet noticeable.

Powdery mildew infection is often associated with host cell death, in particular exemplified as the final consequence of resistance (*R*) gene-mediated fungal growth arrest [23]. Owing to a potential shift in the ratios of plant to fungal genomic DNA, host cell death responses may interfere with the qPCR-based quantification of powdery mildew pathogenesis. To assess this possibility, we repeated the qPCR time course with representative time points including two Arabidopsis mutants that exhibit powdery mildew-triggered cell death responses. Since no canonical (isolate-specific) cell death-associated *R* gene response has been described for the Arabidopsis-powdery mildew patho-systems [2] we took advantage of two induced mutants (*edr1* (*enhanced disease resistance1*) and *pmr4* (*powdery mildew resistant4*)) in the Col-0 genetic background that trigger local powdery mildew-induced host cell death at the post-penetration stage [24,25]. We validated the occurrence of confined powdery mildew-triggered cell death around fungal infection sites in our conditions by Trypan Blue staining (data not shown). Results from the qPCR assay indicate that the *pmr4* mutant supports similar fungal biomass as the *mlo2* mutant, while fungal biomass seems to be higher (intermediate between Col-0 and *mlo2*/*pmr4*) in case of the *edr1* mutant (Figure 1C and Additional file 2: Figure S2B). This finding is in accordance with previous reports stating a similar reduction in hyphal length for *mlo2* (=*pmr2*) and *pmr4* in comparison to the wild type [7], while the *edr1* mutant permits extensive hyphal growth (similar to wild type), but no conidiation [24]. Thus, the qPCR assay faithfully reflects fungal development even in the context of powdery mildew-triggered host cell death. We can, however, not exclude the possibility that a somewhat reduced plant DNA yield owing to localized cell death responses results in a slight overestimation of fungal biomass in these instances.

### Spore counts of *G. orontii*

Spore formation is a widely used surrogate to determine susceptibility of Arabidopsis to another obligate biotrophic pathogen, the oomycete *Hyaloperonospora arabidopsidis*[14,18]. We therefore adopted this technique for quantification of the reproductive success of *G. orontii*. First macroscopically visible powdery mildew symptoms were usually observed on *eds1* seedlings at 5 dpi and wild type seedlings at 6 dpi as previously described [20]. No macroscopic symptoms were detected on *mlo2* or *mlo2 mlo6 mlo12* mutant plants [3,9]. The Col-0 ecotype is already very susceptible to *G. orontii* infection and from 7 dpi onwards no clear differences to *eds1* could be macroscopically detected anymore. We therefore scored spore abundance at 6 dpi. After harvesting by centrifugation (see Methods for details), conidia were counted using a haemocytometer (Figure 2E and F). *G. orontii* conidiospores could clearly be detected as approximately 35 μm long and 18 μm wide ellipsoid structures that were easily distinguishable from contaminating particles. Size and appearance of spores matched earlier reports on *G. orontii*[2,20]. As expected, no conidia were detected in any isolation from *mlo2 mlo6 mlo12* seedlings. On *mlo2* seedlings we observed significantly reduced numbers (10%-15%) of spores relative to the wild type (Figure 2G and Additional file 2: Figure S2C). This proportion exceeds, however, a previous report using conidiophore counts at the same time point [3], probably due to the averaging effect on tissue types of different susceptibility, some of which are deliberately excluded from penetration and conidiophore counts. From *eds1* seedlings we could repeatedly isolate twice the amount of conidio-spores relative to the wild type (Figure 2G and Additional file 2: Figure S2C), although infection phenotypes were difficult to distinguish macroscopically at 6 dpi. The absolute number of isolated conidia varied from 50.000-90.000 spores/g fresh weight for the wild type across three repeated experiments, probably due to differences in the quality of the inoculum and/or inoculation density. The assay has a high dynamic range of four to five orders of magnitude (0 to about 120.000 spores/g fresh weight; Figure 2G and Additional file 2: Figure S2C) and thus has the potential to reveal even small differences in susceptibility between genotypes.

## Conclusions

Here we present two new protocols for the quantification of the powdery mildew disease on Arabidopsis that each yields reproducible data over a broad range of infection phenotypes (Figure 3; Table 2). Although we used mutants with rather extreme powdery mildew infection phenotypes for the establishment of the procedures, we are confident that the techniques are also useful for the quantification of more subtle differences, although such instances may require a higher number of experimental replications. For the qPCR-based method we have developed efficient and specific primer combinations for the amplification of both host and pathogen DNA. The quantified amount of *G. orontii* relative to Arabidopsis genomic DNA correlated well with the amount of fungal biomass. Fungal development was reflected accurately across both time and different genotypes and could also be determined upon the occurrence of localized powdery mildew-induced host cell death. In our conditions, 5 dpi was determined to be the optimal time point for comparative analysis among genotypes, but the method can also be used to resolve kinetics of powdery mildew pathogenesis (Figure 1B and Additional file 2: Figure S2B and Additional file 3).

**Table 2:** Comparison of methods to assess powdery mildew infection

	**Macroscopic categorization**	**Host cell entry counts**	**Conidiophore counts**	**Hyphal area quantification**	**qPCR**	**Conidia counts**
**Assay type**	semi-quantitative	quantitative	quantitative	quantitative	quantitative	quantitative
**Staining required?**	no	yes	yes	yes	no	no
**Microscopy required?**	no	yes (hundreds of interaction sites)	yes (multiple colonies)	yes (multiple colonies)	no	yes
**Stage of patho-genesis scored**	late (conidiation)	early (host cell entry)	late (conidiation)	early to late (host cell entry to conidiation)	middle to late (hyphal expansion to conidiation)	late (conidiation)
**Importance of equal inoculation density**	high (no normalization)	low (internal normalization)	low (internal normalization)	low (internal normalization)	low (internal normalization)	medium (averaging effect of scoring multiple plants at once)
**Suitable for high-throughput analysis**	yes	no	no	no	yes	yes
**Reference**	[6,8]	[3,9]	[3,7,8]	[10-12]	this publication	this publication

We have also adapted a spore count assay to the requirements of powdery mildew research. This assay has a wide dynamic range and accurately and reproducibly determined differences in conidiation by the pathogen (Figure 2G and Additional file 2: Figure S2C). Both assays yielded comparable results for the susceptibility of the genotypes tested and can easily be adapted to other Arabidopsis-infecting powdery mildew species and other plant-powdery mildew patho-systems. Both methods require similar amounts of time for data generation. The qPCR method is intrinsically more cost-intensive but allows simultaneous DNA extraction and PCR analysis of many samples. Spore counts are more cost-effective and, owing to the lower number of practical steps involved, offer less possibilities for experimental error (Figure 3).

Currently, powdery mildew infection is either assessed semi-quantitatively by coarse categorization and thus macroscopic symptoms or quantitatively by microscopic penetration rate counts at early and/or conidiophore counts at late stages (Table 2). The latter methods require staining, mounting and microscopy and are therefore time-consuming and labor-intensive. Both assays introduced here are quantitative and are applied at late stages of infection when differences between genotypes are more pronounced. They do not require staining, are easy to perform and can generate quantitative data for larger numbers of genotypes (Table 2). In addition, the spore count assay is based on many seedlings rather than few mature plants, which offers the advantage of an averaging effect, lowering the possibility of experimental outliers. These assays can therefore complement and overcome limitations of currently used methods of powdery mildew quantification. They might be particularly suitable for the assessment of larger numbers of different mutant lines, e.g. in the course of follow-up analyses of genes identified by –omics approaches.

## Methods

### Plant material and inoculations

In this study we used the *Arabidopsis thaliana* Col-0 genotype and the *edr1*-1 [24], *eds1-*2 [21], *pmr4*-1 [7], *mlo2-*6 single and *mlo2-*5 *mlo6-*2 *mlo12-*1 triple mutants [3] in the Col-0 genetic background. Approximately 100 seeds were sown per pot of soil substrate and five pots were used per genotype. After stratification for 2 d at 4°C in darkness plants were grown for 18 d at a day/night cycle of 10/14 h in a light chamber with 22°C/20°C day/night temperature and a relative humidity of 60%.

The *G. orontii* isolate MPIPZ was propagated on four week old *eds1*-2 plants and conidia were used at 14–21 dpi. Inoculations were performed in a simple 80 cm high cardboard settling tower whose opening was covered with a 80 μm nylon mesh [8]. The tower contained up to nine pots per inoculation. We therefore had to use three consecutive rounds of infection. In each round, pots of all genotypes were included. A fine paint brush was used to harvest conidia from four heavily infected leaves and to separate the conidia by brushing them through the nylon mesh. Inoculation density was approximately 750 spores/cm^2^. Newly inoculated seedlings were then returned to the growth chamber.

### Staining and microscopy

For the visualization of fungal structures, seedlings were harvested at indicated time points and destained and stored in ethanol:glacial acetic acid 3:1 (v/v). Fungal structures were stained with Coomassie Brilliant Blue as described previously [11] and brightfield images obtained using a AxioImager.A2 system with an AxioCam HRc (Zeiss, Jena, Germany). The experiment was repeated twice and 5–10 images were analyzed per replicate, genotype and time point.

### Genomic DNA extraction

Ten seedlings per genotype were harvested across pots and frozen in liquid nitrogen. Genomic DNA was extracted essentially as previously described [16]. Approximately 15 1 mm and 100 mg 0.2 mm diameter glass beads were added and the frozen material was disrupted in a MM400 mixer mill (Retsch, Haan, Germany) for 2x1 min at 30 Hz. Subsequently, 300 μl lysis buffer (2.5 M LiCl, 50 mM Tris–HCl, 62.5 mM Na_2_-ethylenediamine tetraacetic acid (EDTA), and 4.0% Triton X-100, pH 8.0) and an equal volume of phenol:chloroform:isoamyl alcohol (25:24:1 v/v, Carl Roth, Karlsruhe, Germany) were added and samples were homogenized for 30 s at 30 Hz in the mixer mill. After centrifugation (5 min, 16.000 g) the supernatant was recovered and the genomic DNA was precipitated by the addition of two volumes of 100% ethanol, incubation for 15 min at −20°C and another round of centrifugation. The DNA pellet was washed with 70% ethanol, air dried and resuspended in Millipore water. DNA quality and concentration were inspected on a Nanodrop system (Thermo Scientific, Bonn, Germany).

### Quantitative real-time PCR

For qPCR 15 μl samples were prepared using the Brilliant Sybr Green QPCR Reagent Kit (Stratagene, Waldbronn, Germany) according to the manufacturer’s protocol. The *Taq* DNA polymerase provided was replaced by another *Taq* polymerase (Ampliqon, Odense, Denmark) and the corresponding standard buffer. We used a final primer concentration of 0.4 μM and three technical replicates per sample. qPCR was carried out according to the following protocol: denaturation at 95°C for 3 min, 40 repeats of 95°C for 20 s, 61°C for 20 s and 72°C for 15 s. A melting curve analysis was conducted from 55°C-95°C in 0.5°C steps and 10 s dwell time to confirm the amplification of single amplicons. Additionally, amplicon size and identity were confirmed on a 2% agarose gel and by DNA sequencing, respectively. The ratio of *G. orontii* to Arabidopsis genomic DNA was calculated using the ΔΔCt method [26].

### Spore counts

At 6 dpi, three samples of approximately 500 mg of seedlings were harvested per genotype. Five ml H_2_O was added and spores liberated by vortexing for 30 s at maximum speed. The spore solution was filtered through Miracloth (Merck, Darmstadt, Germany) to remove large debris and spores were 4-fold concentrated by centrifugation (5 min, 4000 g). For each sample, spores were counted in eight 1 mm^2^ fields of a Neubauer-improved haemocytometer (Marienfeld, Lauda-Königshofen, Germany) and results were averaged. Finally, spore counts were normalized to the initial weight of seedlings.

## Abbreviations

dpi: days post inoculation; hpi: hours post inoculation; qPCR: quantitative polymerase chain reaction.

## Competing interest

The authors declare that they have no competing interests.

## Authors’ contributions

RW designed and performed the experiments and drafted the manuscript. RP conceived of the study, participated in its design and edited the manuscript. Both authors read and approved the final manuscript.

## Supplementary Material

Additional files 1 **Figure S1. **Documentation of technical details related to the qPCR assay. (A) Primer efficiency calculations for primer sets R189/R192 (red) and R193/R194 (blue). Efficiency was calculated from a 5-fold dilution series. The respective correlation coefficients (R^2^) are indicated. Ct values of *G. orontii* gDNA amplification from the *eds1* time series from 2–6 dpi are presented in green for comparison. (B) Comparison of amplification plots of *G. orontii*-infected Col-0 at 1 (blue) and 5 dpi (green) and the uninfected Col-0 control (red). Raw fluorescence data were exported and used for visualization. Ratios of *G. orontii* to Arabidopsis gDNA were determined by qPCR with primers R189/R192 and R193/R194, respectively. Click here for file

Additional files 2 **Figure S2. **Additional independent replicates of data presented in the main text. (A) qPCR analysis of a time series of powdery mildew infection on Col-0 wild type, *eds1*, *mlo2* and *mlo2 mlo6 mlo2* seedlings. Ratios of *G. orontii* to Arabidopsis gDNA were determined by qPCR with primers R243/R244 and R263/R264, respectively. Bars represent the mean ± standard deviation of three technical replicates from a DNA sample of ten pooled seedlings grown in five different pots (two seedlings/pot used).(B) qPCR analysis of powdery mildew infection on Arabidopsis mutants that show powdery mildew-induced cell death. Representative time points of infection on Col-0 wild type, *eds1, mlo2, pmr4* and *edr1* seedlings were used. Ratios of *G. orontii* to Arabidopsis gDNA were determined by qPCR with primers R189/R192 and R193/R194, respectively. Bars represent the mean ± standard deviation of three DNA samples (each derived from ten pooled seedlings grown in five different pots) with three technical replicates each. (C) Spore counts of indicated genotypes at 6 dpi normalized to seedling fresh weight. Bars represent the mean ± standard deviation of three samples (500 mg of seedlings each) from one experiment counting eight fields/sample. Asterisks indicate statistically significant differences to Col-0 in two-tailed Student’s t-test (p <0,05). Click here for file

Additional files 3 **Figure S3. **qPCR analysis of a time series of powdery mildew infection performed with a second primer set. Samples were harvested at indicated time points from a time series of powdery mildew infection on Col-0 wild type, *eds1*, *mlo2* and *mlo2 mlo6 mlo2* seedlings. Ratios of *G. orontii* to Arabidopsis gDNA were determined by qPCR with primers R243/R244 and R263/R264, respectively. Bars represent the mean ± standard deviation of three technical replicates from a DNA sample of ten pooled seedlings grown in five different pots (two seedlings/pot used). Asterisks indicate statistically significant differences to Col-0 in two-tailed Student’s t-test (p <0,05). Click here for file
