# Supplementary figures and images for: Rapid quantification of plant-powdery mildew interactions by qPCR and conidiospore counts
Source: Plant Methods. 2012 Aug 31;8:35. doi: 10.1186/1746-4811-8-35 (PMC3522566; doi:10.1186/1746-4811-8-35)

**A**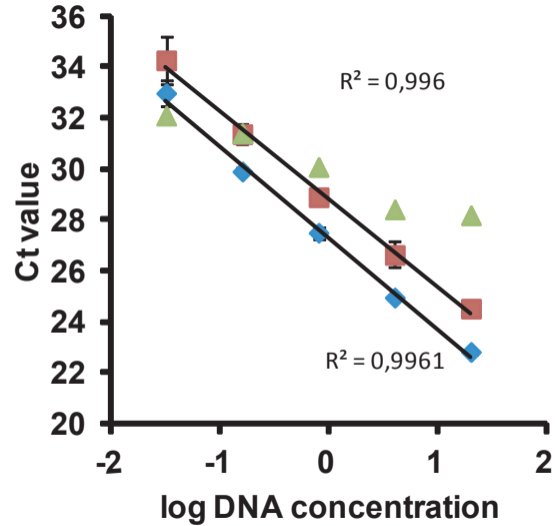**B**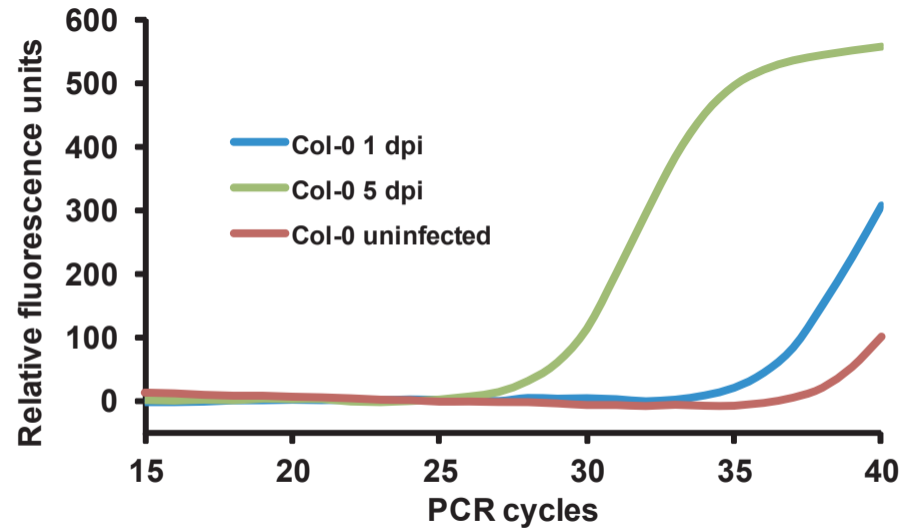

Supplement: Additional files 1 — Figure S1. Documentation of technical details related to the qPCR assay. (A) Primer efficiency calculations for primer sets R189/R192 (red) and R193/R194 (blue). Efficiency was calculated from a 5-fold dilution series. The respective correlation coefficients (R2) are indicated. Ct values of G. orontii gDNA amplification from the eds1 time series from 2–6 dpi are presented in green for comparison. (B) Comparison of amplification plots of G. orontii-infected Col-0 at 1 (blue) and 5 dpi (green) and the uninfected Col-0 control (red). Raw fluorescence data were exported and used for visualization. Ratios of G. orontii to Arabidopsis gDNA were determined by qPCR with primers R189/R192 and R193/R194, respectively. [file 1746-4811-8-35-S1.pdf]

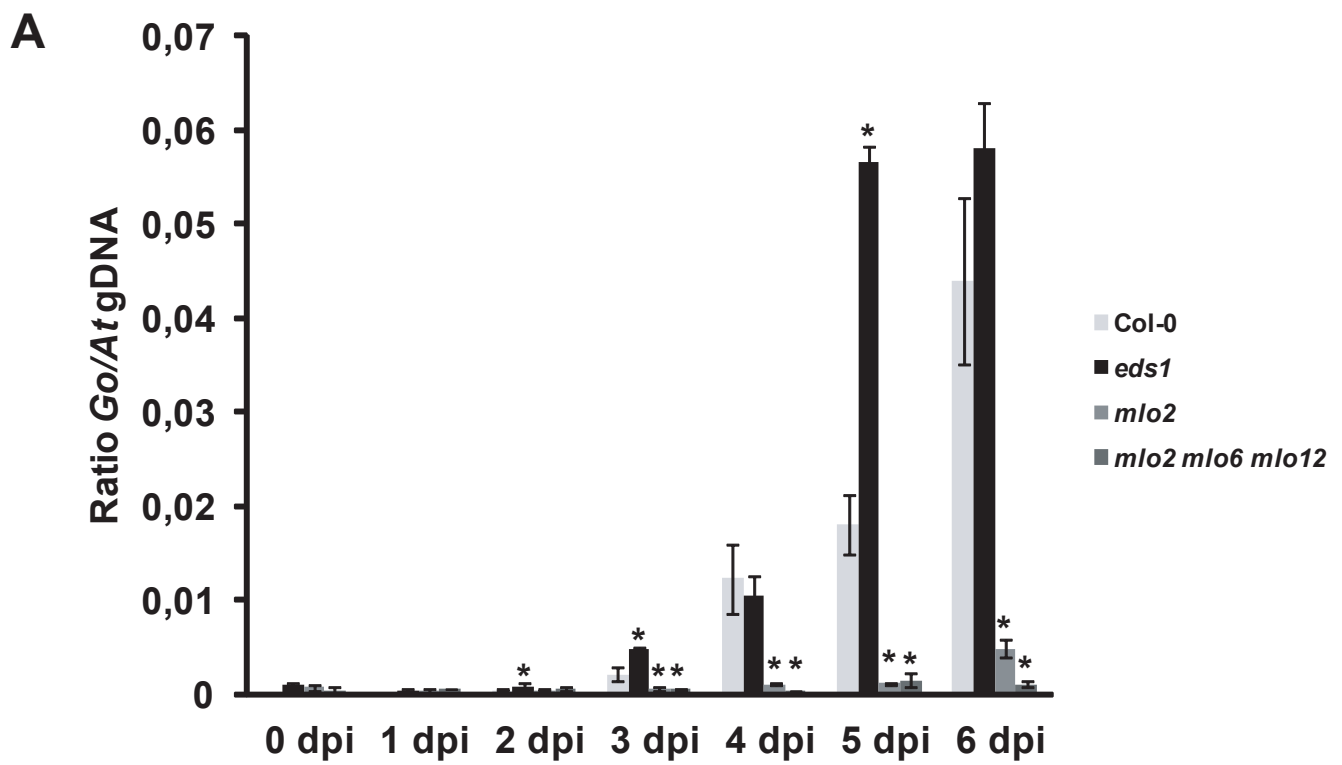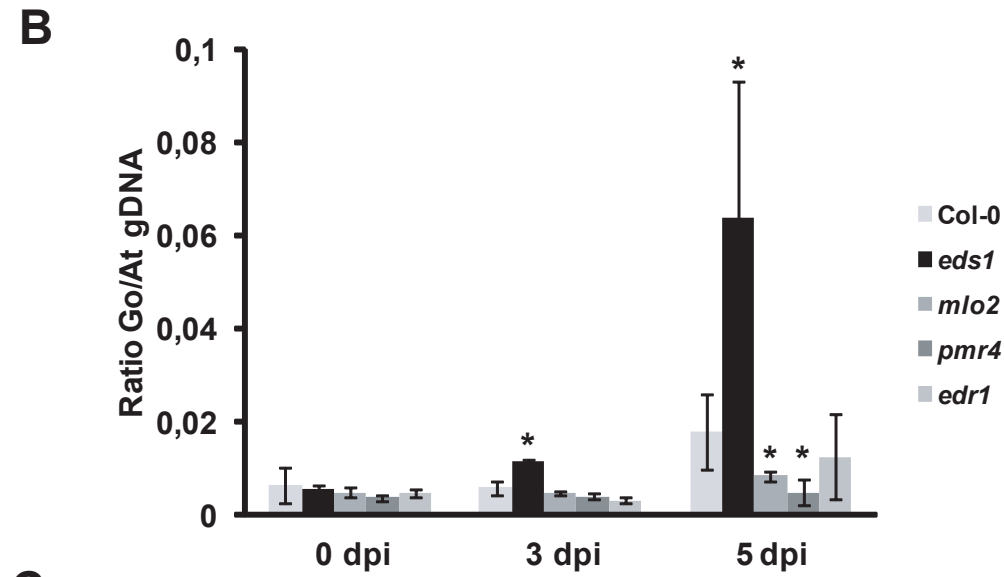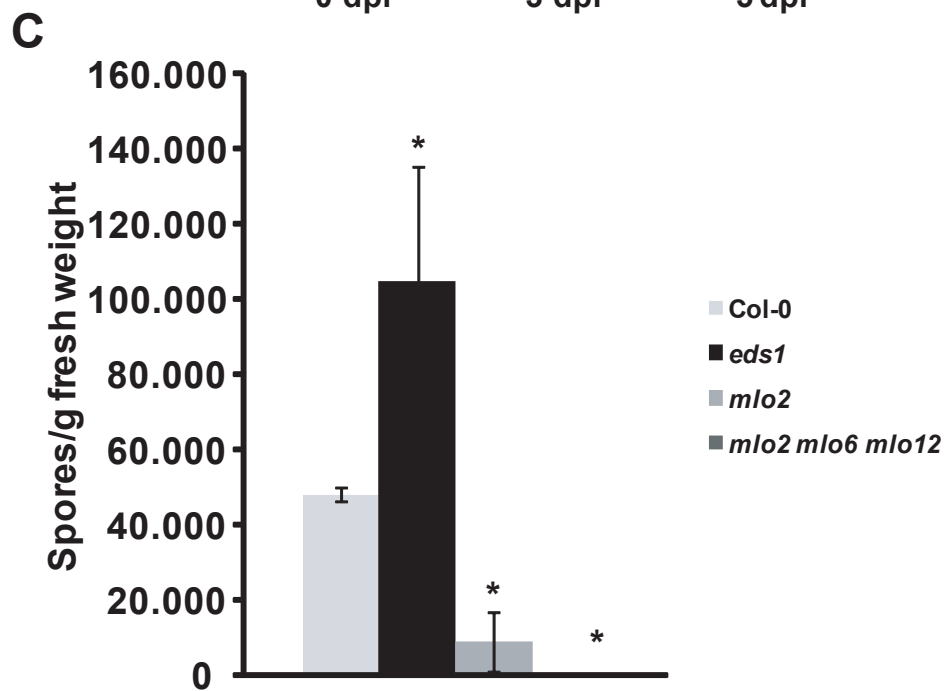

Supplement: Additional files 2 — Figure S2. Additional independent replicates of data presented in the main text. (A) qPCR analysis of a time series of powdery mildew infection on Col-0 wild type, eds1, mlo2 and mlo2 mlo6 mlo2 seedlings. Ratios of G. orontii to Arabidopsis gDNA were determined by qPCR with primers R243/R244 and R263/R264, respectively. Bars represent the mean ± standard deviation of three technical replicates from a DNA sample of ten pooled seedlings grown in five different pots (two seedlings/pot used).(B) qPCR analysis of powdery mildew infection on Arabidopsis mutants that show powdery mildew-induced cell death. Representative time points of infection on Col-0 wild type, eds1, mlo2, pmr4 and edr1 seedlings were used. Ratios of G. orontii to Arabidopsis gDNA were determined by qPCR with primers R189/R192 and R193/R194, respectively. Bars represent the mean ± standard deviation of three DNA samples (each derived from ten pooled seedlings grown in five different pots) with three technical replicates each. (C) Spore counts of indicated genotypes at 6 dpi normalized to seedling fresh weight. Bars represent the mean ± standard deviation of three samples (500 mg of seedlings each) from one experiment counting eight fields/sample. Asterisks indicate statistically significant differences to Col-0 in two-tailed Student’s t-test (p <0,05). [file 1746-4811-8-35-S2.pdf]

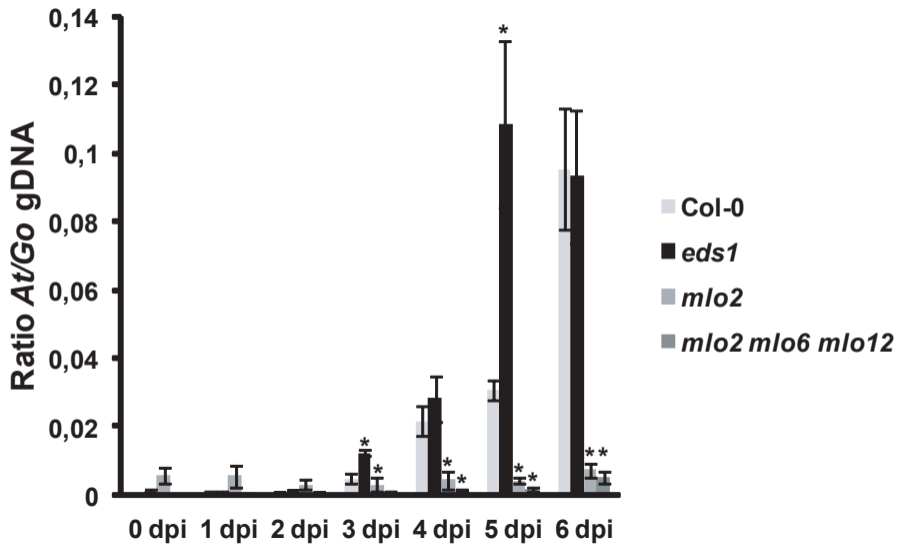

Supplement: Additional files 3 — Figure S3. qPCR analysis of a time series of powdery mildew infection performed with a second primer set. Samples were harvested at indicated time points from a time series of powdery mildew infection on Col-0 wild type, eds1, mlo2 and mlo2 mlo6 mlo2 seedlings. Ratios of G. orontii to Arabidopsis gDNA were determined by qPCR with primers R243/R244 and R263/R264, respectively. Bars represent the mean ± standard deviation of three technical replicates from a DNA sample of ten pooled seedlings grown in five different pots (two seedlings/pot used). Asterisks indicate statistically significant differences to Col-0 in two-tailed Student’s t-test (p <0,05). [file 1746-4811-8-35-S3.pdf]
